# Capacity and willingness to use information technology for managing chronic diseases among patients: A cross-sectional study in Lahore, Pakistan

**DOI:** 10.1371/journal.pone.0209654

**Published:** 2019-01-10

**Authors:** Sadia Iftikhar, Anum Saqib, Muhammad Rehan Sarwar, Muhammad Sarfraz, Mosab Arafat, Qurat-ul-ain Shoaib

**Affiliations:** 1 Department of Pharmacy Practice, Akhtar Saeed College of Pharmaceutical Sciences, Lahore, Punjab, Pakistan; 2 Department of Pharmacy, The Islamia University of Bahawalpur, Bahawalpur, Punjab, Pakistan; 3 College of Pharmacy, Al Ain University of Science and Technology, Abu Dhabi, UAE; 4 College of Pharmacy, University of the Punjab, Lahore, Pakistan; The University of Hong Kong, HONG KONG

## Abstract

**Background and objectives:**

The information technology is a pivotal source of communication between patients and healthcare providers for managing chronic diseases. The objective of this study is to assess the capacity and willingness of patients to use information technology for managing chronic diseases.

**Methods:**

A descriptive, cross-sectional study design was employed. Study was conducted in six tertiary care hospitals of Lahore, Pakistan. The study population consisted of patients aged ≥18 years and diagnosed with a minimum of one chronic non-communicable disease. A structured questionnaire was administered to the study participants for data collection. SPSS was used for data analysis.

**Results:**

Among the 400 respondents, hypertension (39.5%) was the leading chronic condition followed by diabetes (27.5%). Majority of the patients owned a cell phone (90.7%) and had internet access (66.2%). Almost half of the respondents (51.0%) were willing to use text messages; whereas 78.5% and 75.7% of the respondents were reluctant to use video conference and e-mail as a source of communication with healthcare providers. Reason for unwillingness to use e-mail was the patients’ desire to be directly examined by the doctor; whereas unfamiliarity with the use of text message and video conference was the major reason for not using these technologies. Logistic regression analysis revealed that interest in using e-mail to interact with specialist was more among those participants who had good self-reported health (OR = 2.579, 95%CI = 1.276–5.212, *p* = .008), access to internet (OR = 5.416, 95%CI = 2.777–10.564, *p* < .001), and those who owned a cell phone (OR = 12.944, 95%CI = 1.751–95.704, *p* = .012). Interest in using text messages to interact with specialist was more among participants with middle-income group (OR = 2.303, 95%CI = 1.389–3.818, p < .001), residency in close proximity to healthcare professional (OR = 3.529, 95%CI = 2.333–5.339, p < .001), access to internet (OR = 3.253, 95%CI = 2.102–5.033, p < .001) and among those who owned a cell phone (OR = 46.709, 95%CI = 6.335–344.377, p < .001). Interest in using video conference to interact with specialist was more among those participants who had access to internet (OR = 5.840, 95%CI = 2.825–12.069, *p* < .001) and among those who owned a cell phone (OR = 11.177, 95%CI = 1.510–82.725, *p =* .018).

**Conclusion:**

This study concluded that nearly half of the respondents were willing to use text messages; whereas, majority was reluctant in using video conference and e-mail as a source of communication with healthcare providers. Most of the respondents who were located farther from the health care provider were willing to use video conferencing in case it could save more than 60 minutes of their time.

## Introduction

Digitalization has played a pivotal role in providing healthcare services to the patients [1]. Numerous sufferers of chronic diseases seek health related advice through information technology [2]. Health care beneficiaries have shown substantial concern regarding the use of information technologies such as electronic mail (e-mail), text messages and video conferences in facilitating the management of chronic non-communicable diseases such as hypertension, diabetes mellitus and vascular disease [3–5]. The interaction between patients and healthcare provider can be considered as a motivating factor for using information technology and thus responsible for patients’ well-being [6–8]. It is evident that patients seek information technology due to feeling of empowerment and to continuously engage with the healthcare provider [8, 9]. The utility of telecommunication devices for the purpose of communication with the healthcare providers is strongly associated with the patients’ well-being. The advent of technology has made the healthcare resources and health-related information accessible for those patients who are living in remote areas. It has widely improved the quality of health services and lowered the economic burden of disease [3, 10–12]. The electronic devices and application software’s have made the communication easiest and fastest for both the patients and healthcare providers. For instance, sending text messages through mobile phones is the best suited way for reminding patients about their therapeutic regimen or other prescribed healthy activities. Moreover, these devices enable the patients to ask brief queries from their healthcare provider [3]. Scheduling appointment with doctors and visiting them might be inconvenient for the patients, particularly for the residents of remote areas. This problem can be resolved through video conference that can act as a substitute for face-to-face encounters or private interactions between patients and provider [13]. In modern era, health providers strongly influence the health information seeking behavior of patients by showing substantial interest in providing the health related information to the sufferers of chronic diseases through emails, mobile phones and video conferencing [4, 5, 14–18].

The models of health services utilization are the significant markers in demonstrating the accessibility and coverage of healthcare resources by defining variables, establishing relationship between them and evaluating various programs and policies [19, 20]. The Andersen behavioral model is a conceptual framework especially designed for highlighting and validating the determinants responsible for the utilization of healthcare services [19]. This model shows the utilization of health services as a function of three major dynamics which mainly include need, predisposing and enabling factors. Need factors encompass both the perceived and actual healthcare needs of a patient. Predisposing factors can be considered as exogenous because these primarily involve socioeconomic status, racial differences, demographic variables, and health beliefs. Thus, a person is more likely to use healthcare resources if he perceives health services as a cornerstone in the provision of efficient therapy. Finally, the scope of enabling factors comprehends several items like health insurance status, family support, accessibility to health services and societal beliefs, etc., [19]. According to motivational model, individual’s attitude towards information technology, gap between patient-physician relationship and technology associated anxiety have a great impact on the patients’ willingness to use information technology [20].

Globally, Pakistan stands at third position among the countries in which telecommunication market is growing unprecedentedly due to the availability of mobile phones, internet service providers, and application software’s [21]. A report published by Pakistan Telecommunication Authority (PTA) in 2018 revealed that approximately 150 million citizens of Pakistan are the subscribers of cellular services [21]. Thus, Pakistan is on the verge of becoming a strong digital economy and this will have a positive influence on the standards of living and health outcomes of the local masses in all over the country.

In Pakistan, most of the patients with chronic diseases are dealt in primary care settings by the healthcare professionals who generally practice in built-up areas [22]. Various healthcare organizations have taken the initiative of introducing secure email communication between patients and health providers [22]. This initiative is associated with some fears for both patients and health providers. For instance, the health providers are concerned about the fact that a large number of emails would be bombarded to them from their patients; whereas the patients worry that their communication might be intercepted or read by certain people who are not authorized to do so [23, 24]. Distance to specialists and the financial constraints are the considerable barriers in providing optimal care to the patients, particularly for the patients who live away from healthcare providers [25, 26]. The use of information technology in facilitating the delivery of health care service is not as much reputable in Pakistan as in various European countries. It is due to multifaceted issues in the establishment of healthcare system and the use of e-mails and video conferencing in the management of chronic diseases. In a country like Pakistan, where almost half of the population is illiterate and over two thirds (67%) of the population reside in rural areas [27], lack of education and poor socioeconomic status serve as contributing factors towards decreased capacity and willingness to use information technologies. Thus, the objective of this study is to assess the capacity and willingness of patients to use information technology for managing chronic diseases.

## Methods

### Study design

A descriptive, cross-sectional study was performed to analyze the capacity and willingness of patients to use information technology to facilitate the delivery of health care services for non-communicable diseases in Lahore, Punjab, Pakistan.

### Study settings

The study was conducted in Lahore district, which is capital of the Punjab province of Pakistan. Lahore is the 2^nd^ most populous city of Pakistan and 32^nd^ most populous city in the world with an approximate population of 15,245,000 people. Lahore has a total of 48 private, 17 public, and 1 military hospital [28]. This study was conducted in six different tertiary care hospitals of Lahore (1: Akhtar Saeed trust hospital, 2: Bahria international hospital, 3: Farooq hospital, 4: Doctors hospital, 5: Surgimed hospital, and 6: Jinnah hospital). Hospitals were chosen by random sampling technique for the collection of data. On an average 6,000 patients of all ages visited these hospitals on daily basis. These tertiary care hospitals were similar in terms of staff and scope of services, and consequently physicians followed the same prescribing practices. Moreover, the patient population was almost similar in all the selected tertiary care hospitals. Thus, the random selection of patients from these six hospitals was unlikely to cause significant bias.

### Study population and sample size

The study population consisted of patients aged ≥18 years and diagnosed with a minimum of one chronic non-communicable disease (i.e., hypertension, diabetes, etc.). The minimum sample size calculated for this study was 385 [Eq 1] [29].

n=Nx/((N−1)E2+x) Eq 1

Where N is the population size, x is the CI and E is the margin of error.

With an added contingency of 5% for non-response and inappropriate responses, the final sample was calculated to be 400 patients.

### Data collection and outcome variables

The data was collected from 400 patients (aged 18–65 years) during the period of 1^st^ December, 2017 to 15^th^ February, 2018. For the purpose of this study, investigators designed a questionnaire by thoroughly reviewing the available literature from relevant published studies [3, 11, 12, 14, 30, 31] [S1 Appendix]. Content and face validation was conducted on the initial version of the questionnaire. The opinions of two experts served as basis for content validation. The experts expressed their views related to the importance and relativity of the content. Efforts were made to develop a questionnaire that was brief and simple. The investigators made adjustments and administered the questionnaire to a small group of 50 patients. The proposed changes were included in the questionnaire. SPSS version 21.0 was used for calculation of reliability coefficients. Internal consistency was measured by Cronbach’s alpha, while reproducibility was evaluated using intraclass correlation for each item in the availability and willingness scales, with acceptable values of ≥0.6. Calculation for Cronbach’s alpha was set at 0.70 for availability, and 0.73 for willingness section.

The questionnaire had three sections; first section focused on the patients’ characteristics (demographic, socioeconomic and health-related), second on the availability of equipment’s and third section was related to the willingness to use information technologies (i.e., e-mail, text message and video conference). Furthermore, distance from specialist or clinic was an important parameter considered for this study. The investigators who collected the data were properly trained in terms of approaching patients, securing their permission prior to data collection and administering the questionnaire.

#### Capacity and willingness to use information technologies

Respondents were asked about their capacity and willingness to use information technologies (e-mail, text messages, video conferencing) for communicating with the healthcare providers. If the respondents had capacity but they were unwilling to use information technology then the reason of their unwillingness was explored through open ended questions. A hypothetical question was asked from the respondents about the time that would be saved if they use video conference for seeking the expertise of healthcare providers.

#### Proximity to specialist care

The respondents were classified in to two categories based on their proximity to the specialist care. Respondents who had specialist care close to their place of residence were termed to be “in close proximity”; whereas the remaining were termed to be living “farther” from specialist care.

#### Other variables

On the basis of diagnosis, respondents were classified as “those having single chronic condition” and “those having multiple (more than one) chronic conditions”. The body mass index of study participants was determined thorough their self-reported weight and height, by adjusting for self-reported data [32].

### Data analysis

Data was analyzed by using Statistical Package for Social Sciences (IBM SPSS Statistics for Windows, version 21.0, Armonk, NY: IBM Corp.). The characteristics of the survey respondents (demographic, socioeconomic and health-related) were compared across residence locations through chi-squared analyses. Furthermore, the effect size such as Cramer’s V was carried out to represent the strength of association from chi-squared analyses. Reasons regarding un-willingness to use information technologies were also explored and presented. Between socio-demographic variables and outcomes of interest (i.e., capacity and willingness to use information technology), binary logistic regression modeling was used to examine their association. We adjusted the models for characteristics as described in the behavioral model of health service utilization [19] and motivational model [20], which are the frame works for factors that may be related to participants’ interest in electronic technologies. These variables were considered as baseline characteristics of current quality of health care, and attitude toward new technologies. In addition, participant attitude was determined to find the time that would be saved to persuade them to use video conferencing for specialist visit (i.e. <30minutes, 31-60minutes, >60minutes or “don’t know”) rather than opting for face to face visit. Moreover, results of logistic regression analysis were expressed as Odds Ratio (OR) accompanied by 95% CI and a p-value <0.05 was used considered to be statistically significant.

### Ethics approval and consent to participate

The ethical approval was obtained from the Pharmacy Research Ethics Committee (PREC) at the Akhtar Saeed College of Pharmaceutical Sciences (Reference: 13-2017/PREC, dated November 19, 2017). Before conduction of the study, permission was obtained from the hospital administrators to proceed with the study. The purpose of study was explained to every patient and their verbal consents were audio recorded prior to study. Written consent was not possible for most of the respondents because either they were illiterate or they had problems in reading and/or signing the consent document.

## Results

### Characteristics of the respondents

Out of a total 469 patients, 400 completed the survey (response rate = 85.3%). Just over half of the respondents were females (n = 209, 52.2%) and 41.2% of the participants (n = 165) were aged 40–64 years. Over three quarters of the respondents were married (n = 310, 77.5%) and 46.0% had acquired tertiary education. Hypertension (n = 158, 39.5%) was the most common chronic condition followed by diabetes (n = 110, 27.5%) and cardiovascular diseases (n = 103, 25.7%). A detail description of patient characteristics is presented in Table 1.

**Table 1:** Characteristics of the participants.

Variables		Close proximity to specialist (n = 221, %*)	Farther from specialist (n = 179, %)	Total (n = 400, %)	*X*^*2*^	*Cramer’s V*	*P*
Gender	Male	99 (44.8)	92 (51.3)	191 (47.7)	1.727	0.066	.189
	Female	122 (55.2)	87 (48.6)	209 (52.2)			
Age (years)	18–39	54 (24.4)	70 (39.1)	124 (31.0)	9.954	0.158	**.007**
	40–64	100 (45.2)	65 (36.3)	165 (41.2)			
	>65	67 (30.3)	44 (24.5)	111 (27.7)			
Civil status	Single	48 (21.7)	42 (23.4)	90 (22.5)	0.173	0.021	.678
	Married	173 (78.2)	137 (76.5)	310 (77.5)			
Educational level (years)	Primary (≤10)	54 (24.4)	44 (24.5)	98 (24.5)	1.018	0.050	.601
	Secondary (11−13)	61 (27.6)	57 (31.8)	118 (29.5)			
	Tertiary (≥14)	106 (47.9)	78 (43.5)	184 (46.0)			
Annual income	Lower income group (PKR0−299,999)	80 (36.1)	67 (37.4)	147 (36.7)	0.820	0.045	.664
	Middle-income group (PKR300,000−999,999)	64 (28.9)	57 (31.8)	121 (30.2)			
	Higher-income group (PKR≥1,000,000)	77 (34.8)	55 (30.7)	132 (33.0)			
Residence	Urban	126 (57.0)	79 (44.1)	205 (51.2)	6.566	0.128	**.010**
	Rural	95 (42.9)	100 (55.8)	195 (48.7)			
Chronic condition	Hypertension	91 (41.1)	67 (37.4)	158 (39.5)	7.889	0.140	**.048**
	Diabetes	67 (30.3)	46 (25.6)	110 (27.5)			
	Cardiovascular diseases	45 (20.3)	58 (32.4)	103 (25.7)			
	Other	18 (8.14)	11 (6.14)	29 (7.3)			
Self-reported health	Good	21 (9.5)	24 (13.4)	45 (11.2)	3.942	0.099	.139
	Moderate	106 (47.9)	95 (53.0)	201 (50.2)			
	Poor	94 (42.5)	60 (33.5)	154 (38.5)			
Smoking	Occasionally	24 (10.8)	33 (18.4)	57 (14.2)	4.784	0.109	.091
	Daily	65 (29.4)	51 (28.4)	116 (29.0)			
	Never	132 (59.7)	95 (53.0)	227 (56.7)			
Obesity	Yes	72 (32.5)	68 (37.9)	140 (35.0)	1.272	0.056	.259
	No	149 (67.4)	111 (62.0)	260 (65.0)			

*Percentages are given with respect to total sample size in respective column.

### Capacity and willingness to use information technology

More than half of the respondents (n = 265, 66.2%) had internet access and most of the respondents (n = 363, 90.7%) owned a cell phone. A large number of respondents were reluctant to use video conference (78.5%) and e-mail (75.7%). Almost half of the respondents (n = 204, 51.0%) showed willingness to use text messages to facilitate the delivery of health care services. Almost three quarter (n = 135, 75.4%) of the respondents who were present farther from a specialist wanted to use video conferencing if it could save more than 60 minutes of their time (Table 2).

**Table 2:** Capacity and willingness to use information technology.

Variables		Close proximity to specialist (n = 221, %*)	Farther from specialist (n = 179, %)	Total (n = 400, %)	*X*^*2*^	*Cramer’s V*	*P*
**Availability of equipment**							
Have access to internet	Yes	165 (74.6)	100 (55.8)	265 (66.2)	15.624	0.198	**< .001**
	No	56 (25.3)	79 (44.1)	135 (33.7)			
Own a cell phone	Yes	208 (94.1)	155 (86.5)	363 (90.7)	6.672	0.129	**.010**
	No	13 (5.9)	24 (13.5)	37 (9.25)			
**Willingness to use technologies**							
Video call	Yes	50 (22.6)	36 (20.1)	86 (21.5)	0.222	0.024	.638
	No	171 (77.4)	143 (79.9)	314 (78.5)			
E-mail	Yes	55 (24.8)	42 (23.4)	97 (24.2)	0.109	0.017	.741
	No	166 (75.1)	137 (76.5)	303 (75.7)			
Text message	Yes	143 (64.7)	61 (34.1)	204 (51.0)	36.885	0.304	**< .001**
	No	78 (35.3)	118(65.9)	196 (49.0)			
**Threshold time saved for use of video conferencing (min)**							
≤30		75 (33.9)	42 (23.4)	117 (29.2)	7.820	0.140	**.020**
31–60		7 (3.2)	2 (1.1)	9 (2.3)			
>60		139 (62.8)	135 (75.4)	274 (68.5)			

*Percentages are given with respect to total sample size in respective column.

Patients’ desire to be directly examined by doctor was the major reason for their lack of willingness to use e-mail. Moreover, unfamiliarity with the use of text message and video conference was cited as the main reason for patients’ reluctance to use these information technologies (Table 3).

**Table 3:** Reasons provided by respondents for lack of willingness to use information technologies.

**E-mail**	Male (n = 145, %*)	**Female (n = 158, %)**	**Total (n = 303, %)**
Don’t know how to use	41 (28.3)	31 (19.6)	72 (23.8)
Don’t like to use	23 (15.9)	19 (12.0)	42 (13.9)
Not private	1 (0.7)	3 (1.9)	4 (1.3)
Don’t think it is useful	27 (18.6)	17 (10.8)	43 (14.5)
Not secure	6 (4.1)	7 (4.4)	13 13 (4.3)
Desire to be directly examined by the doctor	35 (24.1)	71 (44.9)	106 (5.0)
No time to read	12 (8.3)	10 (6.3)	22 22 (7.3)
**Text message**	**Male (n = 106, %)**	**Female (n = 90, %)**	**Total (n = 196, %)**
Don’t know how to use	38 (35.8)	38 (42.2)	76 (38.8)
Don’t like to use	16 (15.1)	13 (14.4)	29 (14.8)
Not private	0 (0.0)	4 (4.4)	4 (2.0)
Don’t think it is useful	24 (22.6)	16 (17.8)	40 (20.4)
Find it annoying to use	14 (13.2)	11 (12.2)	25 (12.8)
Costly	0 (0.0)	2 (2.2)	2 (1.0)
No time to read	14 (13.2)	6 (6.7)	20 (10.2)
**Video conference**	**Male (n = 153, %)**	**Female (n = 161, %)**	**Total (n = 314, %)**
Don’t know how to use	39 (25.5)	54 (33.5)	93 (29.6)
Don’t like to use	25 (16.3)	23 (14.3)	48 (15.3)
Not private	2 (1.3)	5 (3.1)	7 (2.2)
Think it is uncomfortable	31 (20.3)	35 (21.7)	66 (21.0)
Not secure	19 (12.4)	15 (9.3)	34 (10.8)
Costly	2 (1.3)	8 (5.0)	10 (3.2)
No time for it	35 (22.9)	21 (13.0)	56 (17.8)

*Percentages are given with respect to total sample size in respective column.

### Factors associated with willingness to use of information technology

The results of logistic regression analysis examined the association between willingness to use information technologies (i.e., e-mail, text message and video conference) and the independent variables (i.e., gender, age, civil status, educational level, annual income, residence, chronic condition, self-reported health, smoking, obesity, residence location, access to internet and own a cell phone). Results revealed that patients with primary education (OR = 0.403, 95%CI = 0.220–0.738, *p* = .003) and secondary education (OR = 0.323, 95%CI = 0.178–0.587, *p* < .001) were significantly less likely to use email to interact with specialist as compared to patients with tertiary education. Patients with good self-reported health were about 2.6 times more likely to use email (OR = 2.579, 95%CI = 1.276–5.212, *p* = .008) than those with poor self-reported health. While examining the association of smoking status; patients with occasional smoking were significantly less likely to use email to interact with specialist (OR = 0.421, 95%CI = 0.187–0.947, *p* = .036) as compared to patients who never smoke. Patients who had access to internet were 5.4 times more likely to use email (OR = 5.416, 95%CI = 2.777–10.564, *p* < .001) than those who did not have access to internet. On the other hand, patients who owned a cell phone were about 13 times more likely to use email (OR = 12.944, 95%CI = 1.751–95.704, *p* = .012) as compared to those who did not own a cell phone (Table 4).

**Table 4:** Factors associated with willingness to use of information technology.

Variables		E-mail				Text messages				Video conferencing			
		*N (%)*	*OR*	*95% CI*	*P*	*N (%)*	*OR*	*95% CI*	*P*	*N (%)*	*OR*	*95% CI*	*P*
Gender	Male	43 (10.8)	0.834	0.527–1.320	.439	83 (20.8)	0.537	0.361–0.799	**.002**	40 (10.0)	0.913	0.567–1.470	.708
	Female	54 (13.5)	Ref. (1.0)			121 (30.5)	Ref. (1.0)			46 (11.5)	Ref. (1.0)		
Age (years)	18–39	37 (9.2)	1.203	0.679–2.131	.527	63 (20.8)	1.014	0.608–1.693	.957	29 (7.2)	0.950	0.521–1.732	.866
	40–64	31 (7.8)	0.654	0.368–1.164	.149	86 (30.5)	1.095	0.677–1.773	.711	31 (7.8)	0.720	0.402–1.290	.269
	>65	29 (7.2)	Ref. (1.0)			55 (51.2)	Ref. (1.0)			26 (6.5)	Ref. (1.0)		
Civil status	Single	23 (5.8)	1.095	0.637–1.880	.743	47 (11.8)	1.038	0.649–1.660	.876	23 (5.8)	1.2190	0.701–2.120	.482
	Married	74 (18.5)	Ref. (1.0)			157 (13.5)	Ref. (1.0)			63 (15.8)	Ref. (1.0)		
Educational level (years)	Primary	17 (4.2)	0.403	0.220–0.738	**.003**	32 (8.0)	0.342	0.205–0.570	**< .001**	12 (3.0)	0.296	0.150–0.583	**< .001**
	Secondary	17 (4.2)	0.323	0.178–0.587	< **.001**	63 (15.8)	0.771	0.483–1.229	.274	16 (4.0)	0.332	0.180–0.612	**< .001**
	Tertiary	63 (15.8)	Ref. (1.0)			109 (27.5)	Ref. (1.0)			58 (14.5)	Ref. (1.0)		
Annual income	Lower income group	31 (7.8)	0.909	0.515–1.604	.741	72 (18.0)	1.263	0.788–2.026	.332	23 (5.8)	0.598	0.325–1.100	.098
	Middle-income group	36 (9.0)	1.440	0.820–2.530	.205	76 (19.0)	2.303	1.389–3.818	**< .001**	33 (8.2)	1.384	0.786–2.437	.261
	Higher-income group	30 (7.5)	Ref. (1.0)			56 (14.2)	Ref. (1.0)			30 (7.5)	Ref. (1.0)		
Residence	Urban	156 (39.0)	0.962	0.609–1.520	.868	97 (24.2)	1.102	0.744–1.632	.627	156 (39.0)	1.298	0.805–2.093	.285
	Rural	147 (36.8)	Ref. (1.0)			97 (24.2)	Ref. (1.0)			15 (39.2)	Ref. (1.0)		
Chronic condition	Hypertension	42 (10.5)	1.138	0.453–2.858	.783	84 (21.0)	1.216	0.551–2.687	.628	38 (9.5)	1.030	0.409–2.596	.950
	Diabetes	19 (4.8)	0.656	0.245–1.755	.401	46 (11.8)	0.829	0.365–1.883	.655	15 (3.8)	0.535	0.196–1.457	.221
	Heart disease	29 (7.2)	1.232	0.475–3.194	.668	60 (15.0)	1.495	0.654–3.418	.341	26 (6.5)	1.oo7	0.385–2.637	.988
	Other	7 (1.8)	Ref. (1.0)			14 (3.5)	Ref. (1.0)			7 (1.8)	Ref. (1.0)		
Self-reported health	Good	19 (4.8)	2.579	1.276–5.212	**.008**	27 (6.8)	1.500	0.764–2.946	.239	13 (3.2)	1.722	0.820–3.614	.151
	Moderate	44 (11.0)	0.989	0.596–1.642	.966	101 (25.2)	1.030	0.677–1.568	.889	159 (10.5)	0.977	0.581–1.641	.930
	Poor	34 (8.5)	Ref. (1.0)			76 (19.2)	Ref. (1.0)			123 (7.8)	Ref. (1.0)		
Smoking	Occasionally	8 (2.0)	0.421	0.187–0.947	**.036**	17 (4.2	0.269	0.144–0.504	**< .001**	7 (1.8)	0.388	0.165–0.910	**.029**
	Daily	28 (7.0)	0.863	0.514–1.448	.576	50 (12.5)	0.480	0.305–0.755	**.002**	22 (5.5)	0.679	0.391–1.180	.170
	Never	61 (15.2)	Ref. (1.0)			139 (34.8)	Ref. (1.0)			58 (14.5)	ref (1.0)		
Obesity	Yes	31 (7.8)	0.773	0.472–1.266	.306	68 (17.0)	0.835	0.553–1.260	.390	28 (7.0)	0.785	0.470–1.311	.356
	No	66 (16.5)	Ref. (1.0)			111 (27.8)	Ref. (1.0)			59 (14.8)	Ref. (1.0)		
Residence location	Close	54 (13.8)	1.081	0.682–1.714	.741	141 (35.8)	3.529	2.333–5.339	**< .001**	47 (12.0)	1.122	0.695–1.813	.638
	Faraway	42 (12.5)	Ref. (1.0)			62 (15.5)	Ref. (1.0)			38 (9.5)	Ref. (1.0)		
Access to internet	Yes	86 (21.5)	5.416	2.777–10.56	**< .001**	162 (40.5)	3.253	2.102–5.033	**< .001**	78 (19.5)	5.840	2.83–12.07	**< .001**
	No	11 (2.8)	Ref. (1.0)			43 (10.8)	Ref. (1.0)			8 (2.0)	Ref. (1.0)		
Own cell phone	Yes	96 (24.0)	12.94	1.75–95.70	**.012**	203 (51.0)	46.71	6.33–344.37	**< .001**	85 (21.2)	11.177	1.51–82.73	**.018**
	No	1 (0.2)	Ref. (1.0)			1(0.2)	Ref. (1.0)			1 (0.2)	Ref. (1.0)		

Regarding interest in using text messages to interact with specialist, it was found that male patients were significantly less likely to use text messages (OR = 0.537, 95%CI = 0.361–0.799, *p* = .002) as compared to female patients. Similarly, patients with primary education were also significantly less likely to use text messages (OR = 0.342, 95%CI = 0.205–0.570, *p* < .001) than those with tertiary education. Patients with middle-income were 2.3 times more likely to use text messages (OR = 2.303, 95%CI = 1.389–3.818, *p* = < .001) as compared to patients with higher-income. Patients who occasionally (OR = 0.269, 95%CI = 0.144–0.504, *p* < .001) or daily smoke (OR = 0.480, 95%CI = 0.305–0.755, *p* = .002) were significantly less likely to use text messages as compared to those who never smoke. Patients who resided in close proximity to healthcare provider were 3.5 times more likely to use text messages (OR = 3.529, 95%CI = 2.333–5.339, *p* < .001) than those who were residents of faraway proximity. Likewise, patients who had access to internet were about 3.3 times more likely to use text messages (OR = 3.253, 95%CI = 2.102–5.033, *p* < .001) as compared to those who did not have access to internet. Whereas patients who owned a cell phone were about 46.7 times more likely to use text messages (OR = 46.709, 95%CI = 6.335–344.377, *p* < .001) than those who did not own a cell phone (Table 4).

Regarding interest in using video conference to interact with specialist, results revealed that patients with primary (OR = 0.296, 95%CI = 0.150–0.583, *p* < .001) or secondary education (OR = 0.332, 95%CI = 0.180–0.612, *p* < .001) were significantly less likely to use video conference as compared to patients with tertiary education. Similarly, patients who occasionally smoke were also significantly less likely to use video conference (OR = 0.388, 95%CI = 0.165–0.910, *p* = .029) as compared to those who never smoke. Patients who had access to internet were about 5.8 times more likely to use video conference to interact with specialist (OR = 5.840, 95%CI = 2.825–12.069, *p* < .001) than those who did not have access to internet. The patients who owned a cell phone were about 11.2 times more likely to use video conference (OR = 11.177, 95%CI = 1.510–82.725, *p* = .018) as compared to those who did not own a cell phone (Table 4).

## Discussion

The current study set out to determine the capacity and willingness to use information technology for managing chronic diseases among patients. The findings of our study showed that more than two-third of the participants had internet access whereas most of the respondents owned a cell phone. According to an estimate 88.5 million adults had access to internet for gaining health related information by communicating with their health care providers [11]. Despite the presence of internet, majority of respondents were reluctant to use e-mail and video conference for making contact with health care providers. Reason for unwillingness to use e-mail was the patients desire to be directly examined by the doctor; whereas unfamiliarity with the use of text message and video conference was major reason for not using these technologies. It can be attributed to the fact that a large number of people in Pakistan (particularly those residing in the rural areas) are illiterate [33] and unaware about the use of internet. Similarly, communication errors, threat to patient confidentiality, lengthy emails and inability to be examined directly by the healthcare providers might be some of the contributing factors leading to the reluctant behavior of patients [31]. This is in contrast with other studies [3, 10, 34, 35] where patients were ready witted to use email and video conferencing as the sources of communication with healthcare providers. In the current study, statistical analysis revealed that the patients who had access to internet, and owned a cell phone were more interested in using information technology for communicating with healthcare providers. It is quite obvious because the availability of cell phone and internet connection are the prerequisites for using information technology. A study conducted in Washington DC concluded that younger and educated people were more likely to use email as a source of communication [36]. Surprisingly, age was not a determinant of willingness to use information technology in the current study and the respondents with primary education level were more inclined towards its use. It might be due to the reason that most of the people with primary education reside in remote areas of Pakistan and it is quite difficult for them to have a face to face discussion with health care provider. However, the exact reason behind the association of increased willingness and low educational status needs to be explored in Pakistani context.

Our findings revealed that more than half of the respondents were willing to use text messages to facilitate the delivery of health care services. A Cochrane review elucidated that the use of text message helps in improving the chronic conditions like hypertension, diabetes and asthma [37]. Shaw R et. al had also reported positive results associated with the use of text messages as an intervention for weight loss [38]. Use of text messages is convenient because a single server can provide messages to thousands of patients present in various geographic locations [39]. Moreover, as the message is directly conveyed at the cell phone of the patient so the confidentiality is not breached. In the present study, interest in using text messages to interact with specialist was found more among patients with middle-income groups. It has been reported that text messages are substantial source of communication in low income countries due to their quick delivery, decreased cost, safety and decreased intrusiveness [40–43].

Increased distance between the patients and healthcare provider is one of the contributing factors in decreased access to healthcare services [44, 45]. Distance serve as a main barrier for the people who either have reduced access to transport such as elder people or those residing in remote areas. Study findings revealed that the respondents either residing farther from a specialist or in close proximity to a specialist wanted to use video conferencing if it could save more than 60 minutes of their time. It is in line with the findings of other studies where the patients showed willingness to use information technology for their usual or specialized care, particularly in the absence of any healthcare provider in their vicinity or in case when the use of technology helped in saving their time and money [12, 20, 30, 38, 46]. It has been elucidated that due to the associated advantages patients would be ready to pay a small annual fee for the online services provided to them [47].

It is the first study conducted in Pakistan which explores the capacity and willingness of patients to use information technologies; however, this study has some limitations. First, the study was conducted in a single city of Pakistan so the results might not be generalizable to the population of entire country particularly for those residing in rural areas. Second, the study population consisted of only patients; however, views of health care professionals are very crucial and must also be explored.

## Conclusion and recommendations

This study concluded that most of the respondents owned a cell phone and had internet connection. More than half of the respondents were willing to use text messages; whereas majority was reluctant in using video conference and e-mail as a source of communication with healthcare providers. Reason for unwillingness to use e-mail was the patients desire to be directly examined by the doctor; whereas unfamiliarity with the use of text message and video conference was the major reason for not using these technologies.

Most of the respondents who were located farther from the health care provider were willing to use video conferencing in case it could save more than 60 minutes of their time. Future interventional studies can help to find out the impact of information technology on the management of chronic diseases of patients. The results of present study based on behavioral and motivational models of health services utilization have implications for the policy and practice in such a way that these may raise the need of introducing patient oriented educational interventions. The foremost goal of these interventions will be in encouraging the patients to use information technology especially for communication with the healthcare providers. Such initiatives will also be beneficial in making the patients’ attitude positive towards the use of these technologies and overcoming the issues like anxiety and uneasiness associated with the use of technology. Moreover, this study might provoke the healthcare policy makers to take initiatives for introducing communication through information technology with a particular focus on the patients who live in remote areas and where there is unavailability of healthcare providers.

## Supporting information

S1 Appendix Questionnaire of the study. (DOCX)

S1 File SPSS file. (SAV)
