# Supplementary material for: Capacity and willingness to use information technology for managing chronic diseases among patients: A cross-sectional study in Lahore, Pakistan
Source: PLoS One. 2019 Jan 10;14(1):e0209654. doi: 10.1371/journal.pone.0209654 (PMC6328230; doi:10.1371/journal.pone.0209654)
Supplement: S1 Appendix — (DOCX) [file pone.0209654.s001.docx]

**Capacity and willingness to use information technology for managing chronic diseases among patients: a cross-sectional study in Lahore, Pakistan**

**Consent Information sheet**

We are here on behalf of Akhtar Saeed College of Pharmaceutical Sciences. We are conducting a research project on “*Capacity and willingness to use information technology for managing chronic diseases among patients: a cross-sectional study in Lahore, Pakistan”* Your participation is purely based on your willingness. You have the right to choose not to take part in this study. If you choose to take part, you have the right to stop at any time. The information that you provide will be kept confidential by using only code numbers and locking the data.

Based on the understanding of the information that we gave you, are you willing to participate in this study?

Yes   No

**Section A**

**Characteristics of survey patients:-**

1. Gender

Male  Female

1. Age

18-39  40-64  >65

1. Marital status

Single  Married

1. Level of education

Primary  Secondary  Tertiary

1. Annual income

0-3lac  3-10lac  Above 10lac

1. Residence

Urban  Rural

1. Chronic condition

Hypertension  Diabetes

Heart disease  Stroke

1. Additional chronic disease …………………………..
2. Self-perceived health

Good  Moderate  Poor

1. Smoking Status

Occasionally  Daily  Never

1. Obese

Yes  No

**Capacity and willingness to use information technology**

**Section B**

***Availability of Equipment’s***

1. Own a computer with internet access

Yes  No

1. Own a cell phone

Yes No

**Section C**

***Willing to Use Technologies***

1. Video call

Yes  No

1. E-mail

Yes  No

1. Text messages

Yes  No

1. Threshold time saved for use of video conferencing, in

<30  31-60

>60  Don’t know

1. Reasons not to use E-mail

Don’t know how to use  Don’t like to use  Not private

Do not think it is useful  Not Secure

Want to talk to doctor  No time to read

1. Reasons not to use text messages

Don’t know how to use  Don’t like to use

Not private  Do not think it is useful

Find it annoying to use  Costly  No time to read

1. Reasons not to use video-conference call

Don’t know how to use  Don’t like to use  Not private

Think it is uncomfortable  Not Secure  Costly

No time for it

1. Distance from clinic

Close (1-10km)  Faraway (>10km)
